# Molecular mechanisms of ANKH function and regulation in skeletal mineralization

**DOI:** 10.1093/jbmrpl/ziag100

**Published:** 2026-06-16

**Authors:** Nuwanthika Wathuliyadde, Katherine E Willmore, Gregory M Kelly

**Affiliations:** Department of Biology, Western University, London, ON N6A 5B7, Canada; Department of Anatomy and Cell Biology, Western University, London, ON N6A 5C1, Canada; Department of Biology, Western University, London, ON N6A 5B7, Canada

**Keywords:** ANKH, bone, mineralization, inorganic pyrophosphate, inorganic phosphate

## Abstract

Progressive ankylosis homolog (ANKH) is a transmembrane protein essential for regulating bone mineralization through the export of nucleoside triphosphates, primarily adenosine triphosphate (ATP), and citrate into the extracellular matrix. Exported ATP is hydrolyzed by ectonucleotide pyrophosphatase/phosphodiesterase 1 into AMP and inorganic pyrophosphate (PPi). Progressive ankylosis homolog is widely expressed across tissues, where it limits ectopic mineralization of tissues and other roles. Its functional role is particularly prominent in mineralizing cells such as osteoblasts and chondrocytes that maintain the balance between mineral formation and inhibition required for healthy skeletal function. Mutations in ANKH cause 2 main mineralization disorders: craniometaphyseal dysplasia, characterized by progressive craniofacial bone thickening, and calcium pyrophosphate deposition disease (CPPD), marked by crystal deposits causing arthritis-like joint symptoms. Functionally, ANKH operates within a coordinated regulatory axis with ectonucleotide pyrophosphatase/phosphodiesterase 1 and tissue-nonspecific alkaline phosphatase (TNAP) that governs extracellular PPi homeostasis. Although TNAP-mediated PPi hydrolysis contributes negligibly to extracellular inorganic phosphate (Pi) levels, this process remains essential for clearing PPi to prevent excessive inhibition of mineral deposition, thereby preserving the Pi/PPi ratio required for physiological mineralization. Emerging evidence also implicates ANKH in citrate export, influencing bone matrix composition, and mechanical integrity. Further, ANKH expression and function are regulated by multiple signaling pathways including Wnt, tumor necrosis factor-alpha, and FGF, as well as by post-transcriptional modifications, although these regulatory mechanisms remain poorly characterized. Current mouse models, primarily knock-in lines carrying craniometaphyseal dysplasia-associated mutations, only partially recapitulate human phenotypes while comparable models for CPPD-associated mutations remain unavailable. Addressing these gaps by elucidating mutation-specific mechanisms, signaling networks, and developing improved animal models will be critical to advance targeted therapies for ANKH-related mineralization disorders and improve patient outcomes.

## Graphical Abstract

**Graphical Abstract:**
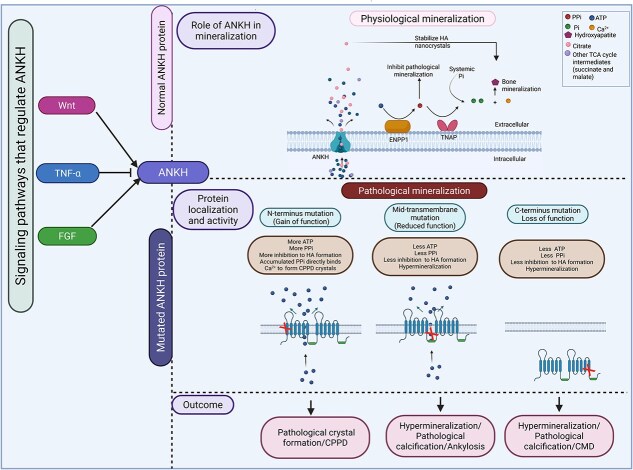

## Introduction

Bone mineralization is a tightly regulated process in which hydroxyapatite (HA) crystals nucleate within matrix vesicles and propagate into the extracellular matrix.^1–3^ The progression of mineralization depends on a finely tuned ratio between inorganic phosphate (Pi), which drives crystal growth, and inorganic pyrophosphate (PPi), which inhibits the mineralization.^1–7^ Progressive ankylosis homolog (ANKH) plays a central role in this process by controlling extracellular levels of PPi, a critical inhibitor of HA.^8–14^ Specifically, ANKH mediates the export of adenosine triphosphate (ATP) into the extracellular space, where it is converted to PPi and AMP by ectonucleotide pyrophosphatase/phosphodiesterase 1 (ENPP1). The generated PPi then modulates mineralization by inhibiting HA deposition.^13^^,^^14^ Although its inhibitory role in mineralization through PPi transport is well established,^8^ recent studies have revealed that ANKH also exports citrate, a key structural component of the HA nanocrystal/collagen complex that contributes to bone matrix quality and mechanical strength.^13^ These findings suggest that ANKH serves a more versatile role in skeletal biology than previously recognized, extending beyond PPi regulation to directly influence the composition and integrity of mineralized tissue. Equally important yet poorly understood is how intracellular signaling pathways regulate *ANKH* expression and activity under both normal and pathological conditions. This regulatory layer likely dictates dynamic functional outcomes of ANKH within mineralizing tissues.^15–22^ In addition, mutations that disrupt ANKH transport function have been directly linked to mineralization disorders, including craniometaphyseal dysplasia (CMD) and calcium pyrophosphate deposition disease (CPPD), but the structure-function relationships underlying these variants remain poorly defined, hindering progress in therapeutic target development.^9–11^^,^^23–31^ Although current in vitro and in vivo models have provided valuable insights into ANKH biology and disease relevance, they fall short of fully recapitulating human phenotypes and the complexity of mineralization regulation.^8^^,^^10^^,^^26^^,^^27^^,^^31^^,^^32^ In this review, we discuss current evidence on the molecular regulation of *ANKH*, its integration into key signaling pathways, and the consequences of disease-associated mutations. We also discuss how emerging experimental model systems are refining our understanding of the contribution of this transporter to physiological and pathological mineralization and delineating the major unresolved mechanistic questions that persist in the field.

## Pi/PPi balance in bone mineralization

Before exploring the mechanisms by which ANKH regulates the Pi/PPi ratio to modulate mineralization, it is essential to first understand the distinct and complementary roles of Pi and PPi in mineralization. Bone mineralization begins within matrix vesicles and collagen fibril gap zones, which provide a confined microenvironment where Pi combines with calcium ions to form an initial metastable amorphous calcium phosphate phase. This amorphous precursor, initially spherical in morphology, progressively transforms through nucleation and stepwise crystal growth into acicular and eventually platelet-shaped nanocrystalline HA.^33^ Inorganic phosphate drives this transition by raising the local calcium-phosphate ion product above the solubility threshold for HA, generating the supersaturation required for nucleation and continued crystal growth, with hydroxyl ions incorporated as an integral component of the developing Ca_10_(PO_4_)_6_(OH)_2_ lattice.^34^ Conversely, PPi acts as a potent inhibitor by adsorbing onto growing HA crystal surfaces, sterically blocking further ion attachment and crystal growth.^1–3^ This inhibition prevents pathological mineralization and ensures tight regulation of mineral deposition. Through this dynamic interplay, the Pi/PPi ratio plays the central metabolic role of a “stencil,” spatially and temporally coordinating mineralization to meet physiological demands, enable tissue repair, and maintain bone strength.^1^^,^^3^^,^^5^ The ratio of Pi to PPi therefore dictates the outcome of this process. Maintaining this balance is essential, as even small disruptions can lead to abnormal bone growth or soft tissue calcification. A dynamic interplay between cellular transporters and enzymes is required to sustain this Pi/PPi ratio and ensure mineralization homeostasis.^5^^,^^7^^,^^24^

## Central role of ANKH in bone mineralization

The Pi/PPi ratio is maintained by an integrated network of local and systemic regulators, with ANKH acting as a central cellular exporter of ATP that provides PPi that works in concert with other pathways to provide spatial and temporal control over mineral deposition.^35–40^ Locally, ANKH increases extracellular ATP levels and functions alongside, ENPP-dependent PPi generation.^41–43^ This PPi is subsequently hydrolyzed by tissue-nonspecific alkaline phosphatase (TNAP) into Pi. Together, these 3 regulators collectively shape the local balance between mineralization inhibitors, such as PPi, and mineralization promoters, such as Pi.^42^^,^^44^^,^^45^ The type III sodium-dependent phosphate cotransporters (PIT1) and (PIT2) further support mineralization by importing Pi into bone cells and matrix vesicles, ensuring sufficient local Pi for HA nucleation and growth.^24^^,^^46^^,^^47^

**Figure 1:**
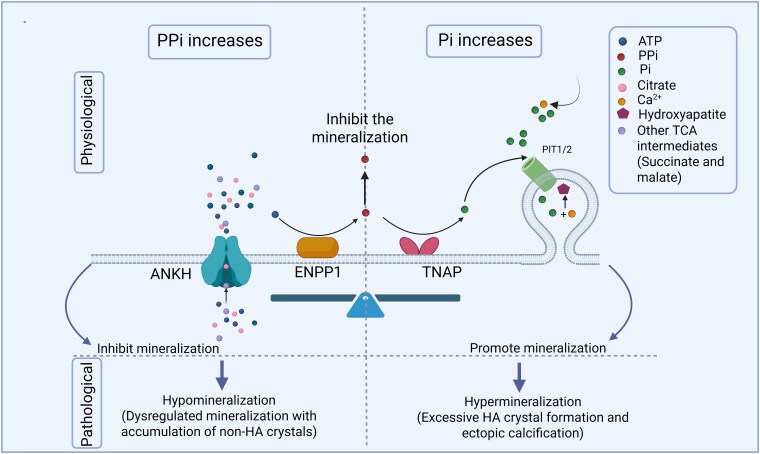
Local regulation of the Pi/PPi balance during bone mineralization. Progressive ankylosis homolog (ANKH) exports ATP, citrate, and to a lesser extent other TCA cycle intermediates (succinate and malate) into the extracellular space. Ectonucleotide pyrophosphatase/phosphodiesterase 1 (ENPP1) hydrolyzes ATP into AMP and inorganic pyrophosphate (PPi), a potent inhibitor of mineralization. Tissue-nonspecific alkaline phosphatase (TNAP) then converts PPi into inorganic phosphate (Pi), which is taken up by osteoblasts and chondrocytes through the sodium-phosphate cotransporters PIT1/2 and, together with Ca^2+^, drives the formation of hydroxyapatite (HA) within matrix vesicles. The relative activities of these transporters and ectoenzymes set the local Pi/PPi ratio (depicted as a balance), which determines whether mineralization is promoted or inhibited under physiological conditions. Disruption of this balance produces pathological outcomes: a shift toward elevated PPi causes hypomineralization with accumulation of non-HA crystals, whereas a shift toward elevated Pi causes hypermineralization and ectopic calcification.

In parallel, systemic hormones, such as vitamin D and PTH, regulate serum phosphate and calcium levels, while circulating inhibitors including fetuin-A and matrix Gla protein prevent inappropriate crystal formation and soft tissue calcification.^19^^,^^21^^,^^22^ Together, these local extracellular matrix factors and systemic cues modulate the Pi/PPi ratio within a physiological window that permits normal mineralization but when perturbed, can give rise to both hyper- and hypomineralization disorders (Figure 1). Rather than acting in isolation, ANKH participates in this broader regulatory network by contributing to extracellular PPi homeostasis, underscoring that therapeutic strategies must consider not only ANKH itself but also its coordinated interactions with other mineralization regulators.

### Integration of ANKH, ENPP1, and TNAP in mineralization control

ANKH is thought to contribute substantially to ATP export in bone, but it operates within an interconnected regulatory system rather than functioning independently. Once exported by ANKH, ATP is hydrolyzed by ENPP1 into PPi and AMP.^13^^,^^14^ Although TNAP-mediated PPi hydrolysis contributes minimally to total extracellular Pi levels, its primary role is to degrade PPi locally at sites of mineralization, thereby shifting the Pi/PPi ratio to permit controlled HA deposition in bone and teeth.^48^ Together, these 3 proteins form the ANKH–ENPP1–TNAP axis, a key regulatory hub in mineralized tissues.^48^ Consistent with this interdependence, bones of Ank^−/−^ mice contain ~75% less pyrophosphate than WT, while bones of Enpp1^−/−^ mice are nearly devoid of pyrophosphate (<2.5%), confirming that these components must act sequentially for normal pyrophosphate deposition in bone.^13^^,^^14^ Balanced activity within this pathway maintains a proper Pi/PPi environment. Even subtle disruptions within this axis can destabilize mineral homeostasis and create pathological outcomes. Understanding how these regulators interact is therefore essential to defining mineralization under physiological and disease conditions (Figure 1).

A notable paradox emerges within this regulatory axis, as mouse models lacking extracellular PPi, including Ank^−/−^ and *Enpp1^−/—^* deficient animals, frequently exhibit reduced rather than enhanced bone mineralization, contrary to what loss of an established inhibitor would predict.^43^ This raises a fundamental mechanistic question, in the absence of PPi, what continues to inhibit mineralization? Several phenomena may help to explain this observation. First, the loss of PPi triggers strong compensatory upregulation of osteopontin (OPN), which binds growing HA crystal surfaces and itself functions as a potent mineralization inhibitor.^43^ In *Ank*^−/−^ and *Enpp1*^−/−^ bones, OPN is markedly elevated and effectively assumes the inhibitory role of PPi, indicating that mineralization is controlled by an integrated network of inhibitors rather than by PPi alone.^43^ Additional inhibitors, including fetuin-A and matrix Gla protein, further contribute to this redundant control system.^19^^,^^21^ Second, the “reduced mineralization” reported in these models refers primarily to bone matrix quality rather than total bone mass; *Ank*^−/−^ and *Ank*^*KI/KI*^ animals typically display increased bone volume but with hypomineralized cortices, smaller and disorganized HA crystals, and impaired mechanical integrity.^26^^,^^27^ Importantly, mineralization is not suppressed in these animals but rather misdirected, as evidenced by extensive ectopic calcification in joints, cartilage, and soft tissues.^8^ Third, ANKH-mediated citrate export is independently required for HA nanocrystal stabilization, so loss of ANKH compromises matrix quality through a PPi-independent mechanism.^14^ Finally, chronic disruption of extracellular PPi homeostasis perturbs osteoblast and osteoclast differentiation, further contributing to the abnormal mineralization phenotype.^26^^,^^27^ Together, these findings reframe the role of PPi, rather than acting as the sole inhibitor of mineralization, PPi functions within a redundant network of inhibitors and matrix regulators, and its loss does not unleash unchecked mineralization but instead disrupts its spatial organization and matrix quality.

Beyond this established function, ANKH also influences mineralization by modulating extracellular citrate levels, which are vital for stabilizing HA nanocrystals and thereby enhancing bone matrix quality and mechanical strength.^13^^,^^49^ In addition to citrate, ANKH has also been shown to mediate the export of other TCA cycle intermediates, including succinate and malate. However, unlike citrate, the specific contribution of extracellular succinate and malate to bone mineralization remains poorly understood, and their export via ANKH may reflect a broader metabolic transport function rather than a dedicated mineralization regulatory mechanism (Figure 1). Moreover, recent evidence from our group has provided deeper insight into the possible broader biological roles of ANKH beyond bone mineralization. Specifically, we identified 2 paralog genes in zebrafish *ankha* and *ankhb*, whereas *ankhb*, which is localized to bone-developing areas, and *ankha*, which is predominantly expressed at the onset of early brain development before bone forms.^32^ The distinct expression of *ankha* in neural tissue is particularly significant when considered alongside clinical observations that some CMD patients who carry mutations in ANKH exhibit cognitive impairments.^50^ These observations are supported by earlier work from Ding and colleagues, who also identified distinct spatial expression patterns for the 2 paralogs, with *ankha* becoming restricted to the head region and *ankhb* localizing to the head, gut, and pharyngeal arches during zebrafish development.^51^ These findings suggest that ANKH is not exclusively a skeletal protein but is also required for normal brain development. While extensive research has characterized the bone abnormalities in individuals with CMD due to ANKH mutations, the link between these mutations and brain development remains poorly understood. It is plausible that ANKH mutations disrupt ATP export during critical windows of early brain development, depriving the developing neural tissue of extracellular purinergic signals essential for neuronal proliferation, migration, and differentiation processes known to be regulated by extracellular ATP.^52^ This suggests that this impaired ATP transport may contribute to the cognitive deficits observed in certain CMD cases. Collectively, these findings position ANKH as a multifunctional transporter whose roles extend beyond skeletal mineralization to include extracellular citrate regulation and neurodevelopmental processes, as supported by the cognitive deficits observed in CMD.

## Signaling pathways and epigenetic regulation of *ANKH* in mineralization

**Figure 2:**
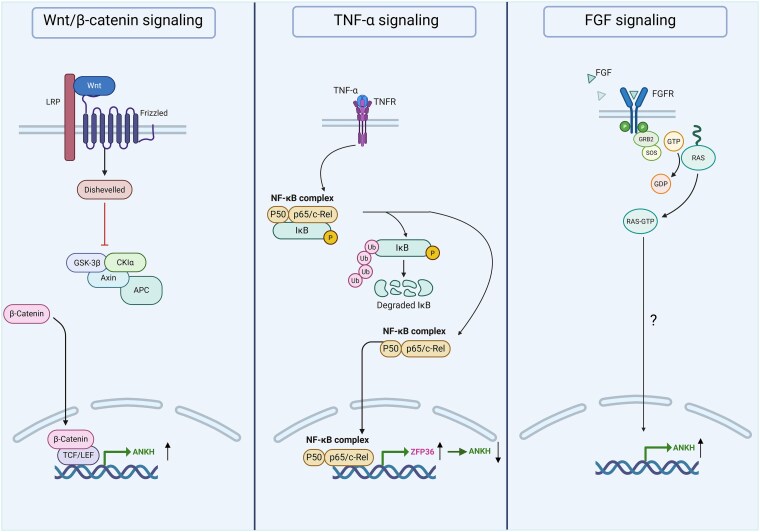
Major signaling pathways regulating progressive ankylosis homolog (*ANKH*) gene expression. (Left) Canonical Wnt/β-catenin signaling: Wnt binding to frizzled and the LRP co-receptor activates disheveled, inhibiting the β-catenin destruction complex (Axin, APC, GSK-3β, and CKIα). Stabilized β-catenin translocates to the nucleus and, with TCF/LEF, upregulates *ANKH* transcription. (Middle) TNFα signaling engagement of the TNF receptor (TNFR) drives IκB phosphorylation, ubiquitination, and proteasomal degradation, releasing NFκB (p50/p65/c-Rel) for nuclear translocation. NFκB induces the RNA-binding protein ZFP36, which destabilizes *ANKH* mRNA and reduces *ANKH* expression. (Right) FGF signaling: FGF binding to its receptor (FGFR) recruits the GRB2–SOS adaptor complex, promoting GDP-to-GTP exchange on RAS. Active RAS-GTP initiates downstream signaling that upregulates *ANKH* expression, although the intermediate effectors linking RAS activation to *ANKH* induction remain undefined. Together, these pathways converge on *ANKH* transcription to fine-tune extracellular pyrophosphate availability and mineralization.

Understanding how *ANKH* expression and activity are regulated by intracellular signaling pathways remains an important yet insufficiently understood aspect of its biology. This regulatory layer likely dictates the dynamic functional outcomes of *ANKH* within mineralizing tissues under both normal and pathological conditions. Current evidence identifies several mechanisms that regulate *ANKH*, including 3 major intracellular signaling pathways Wnt/β-catenin (Wingless/Int-1) family of signaling proteins, tumor necrosis factor-alpha (TNFα), and FGF2 that modulate its transcriptional expression and influence Pi/PPi homeostasis during bone mineralization.^15–22^ In parallel, post-transcriptional control mediated by microRNAs (miRNAs) and long non-coding RNAs, as well as epigenetic modifications, such as DNA methylation, further refine *ANKH* expression patterns in osteoblasts and chondrocytes.^18^^,^^53–56^ These layers of regulation together shape how *ANKH* responds to developmental, inflammatory, and metabolic cues, ultimately determining whether mineralization proceeds in a physiological or pathological direction (Figure 2).

### Wnt/β-catenin pathway regulation of ANKH in mineralization

The Wnt1/β-catenin pathway exerts a crucial role in balancing mineral deposition through its regulation of *ANKH* expression and downstream effects on ATP efflux.^15^^,^^16^ In vascular smooth muscle cells and CKD models, activation of Wnt1/β-catenin signaling markedly upregulates *ANKH* expression, leading to increased extracellular PPi, suppression of osteogenic differentiation, and reduced calcium deposition.^15^^,^^16^^,^^57^ Conversely, *ANKH* knockdown or pharmacological inhibition abolishes these protective effects, establishing *ANKH* as an essential downstream mediator of Wnt1-driven inhibition of vascular calcification.^15^ Moreover, in vivo studies demonstrate that pharmacological Wnt agonism restores *ANKH* expression, elevates circulating PPi levels, and significantly attenuates vascular calcification.^15^^,^^57^ Complementary findings from ankylosing spondylitis fibroblasts show that *ANKH* overexpression inhibits mineralization and promotes β-catenin degradation, while *ANKH* silencing enhances mineralization and activates Wnt/β-catenin signaling.^16^ These findings raise the possibility that *ANKH* may also act upstream of Wnt/β-catenin signaling, rather than functioning purely as a downstream effector.^16^ As the Wnt signaling pathway is highly complex and involved in numerous cellular development and differentiation processes, our understanding of its regulation of *ANKH* remains limited. Further investigation is needed to clarify how other canonical Wnt ligands and non-canonical Wnt pathways influence *ANKH* expression and function, and how this regulation contributes to mineralization control.

### TNFα/NFκB pathway regulation of ANKH in mineralization

The TNFα and NFκB axis represents another major signaling pathway affecting *ANKH*, linking inflammation to bone mineralization.^17^^,^^18^^,^^58^ Under inflammatory conditions, TNFα activation suppresses *ANKH* and *ENPP1* transcription, leading to reduced extracellular PPi and enhanced mineralization.^17^ NFκB contributes to this suppression by inducing *ZFP36* (tristetraprolin), an RNA-binding protein that promotes *ANKH* mRNA degradation. Reduced *ANKH* expression lowers extracellular PPi, which, together with TNF-induced increases in TNAP activity, creates a pro-mineralizing environment.^58^ Moreover, pathways such as stromal cell-derived factor-1 (SDF-1)/CXCR4 signaling acts through NFκB to downregulate *ANKH*, an effect that overexpression of *ANKH* can partially rescue, restoring PPi levels.^59^ However, critical questions remain unanswered, including how the duration of TNF exposure and concentration affects *ANKH* regulation and mineralization. Also, the precise molecular mechanisms by which downregulation of *ANKH* affects bone mineralization through changes in pyrophosphate levels and downstream bone markers remain unclear and require further investigation to fully elucidate these pathways.

### FGF pathway regulation of ANKH in mineralization

Fibroblast growth factor signaling, provides a crucial regulatory layer over *ANKH* expression and the Pi/PPi balance. Activation of FGFR-mediated signaling by basic FGF significantly upregulates *ANKH* expression while concurrently suppressing alkaline phosphatase (*ALP*/TNAP) activity, resulting in a lowered extracellular Pi/PPi ratio. This shift in the mineral microenvironment promotes an anti-mineralizing state.^20^^,^^60^ Evidence from human stem cells derived from exfoliated deciduous teeth demonstrates that bFGF/FGF2 increases *ANKH* mRNA levels within 6-24 h while suppressing *ALP* expression, thus shifting the pericellular Pi/PPi balance toward increased PPi and inhibition of mineral deposition. In contrast, supplementation with ALP rescues the mineralization deficit. The dependence of these effects on FGFR signaling is confirmed by the use of the FGFR inhibitor SU5402, which abolishes both *ANKH* induction and *ALP* repression.^60^ Parallel studies using murine pre-osteoblast MC3T3-E1 cells show that FGF induces both *ANKH* and *ENPP1* expression, suppressing *ALP* and altering PPi metabolism to reduce mineralization.^20^ Together, these data from diverse cell types underscore a conserved role of FGF2–FGFR signaling in modulating phosphate homeostasis and mineralization through coordinated regulation of *ANKH*, *ENPP1*, and *ALP*. While it is established that FGF signaling regulates *ANKH* expression, the detailed intermediate subcellular changes and the exact downstream signaling mechanisms through which FGF modulates *ANKH* remain poorly elucidated.

### Post-transcriptional regulators of ANKH

Post-transcriptional and epigenetic mechanisms play vital roles in regulating *ANKH* expression. As mentioned above (NFκB signaling), tristetraprolin, which promotes deadenylation and decay of target mRNAs, acts as a post-transcriptional regulator similar to miRNAs, which also play critical roles in regulating *ANKH* expression.^58^ There are several other miRNAs that directly or indirectly influence *ANKH* levels and mineralization outcomes. For example, miR-20a directly targets the 3′-untranslated region of *ANKH* in cartilage endplate chondrocytes, reducing ANKH protein expression and extracellular PPi, while miR-17-5p has been shown to regulate *ANKH* indirectly in ankylosing spondylitis fibroblasts.^53^^,^^61^ This repression of *ANKH* reduces extracellular PPi levels, and promotes pathological mineral deposition and calcification.^17^ Epigenetic modifications, including DNA methylation of miRNA promoters and RNA methylation (m6A), further influence miRNA availability and activity in osteoarthritic cartilage, adding another regulatory layer to *ANKH* expression.^56^ Long non-coding RNAs contribute to the regulation of *ANKH* in chondrocytes by modulating critical cellular processes, such as proliferation, differentiation, apoptosis, and extracellular matrix remodeling. The lncRNA, AC008440.5, promotes osteoarthritis progression by sequestering miR-328-3p and preventing it from suppressing its targets *ANKH* and Aquaporin 1, leading to their upregulation, reduced chondrocyte viability, and increased extracellular matrix degradation.^55^

Together, signaling pathways, miRNA regulation and epigenetic modifications collectively alter *ANKH* expression and potentially contribute to mineralization disorders. However, the precise mechanisms through which these regulatory systems influence *ANKH* activity and, in turn, bone or cartilage mineralization remain poorly understood and require further investigation. Disruptions arising from inherited mutations, dysregulated signaling, or altered epigenetic control can impair ANKH function, leading to disease. A deeper understanding of these interconnected regulatory layers is therefore essential for developing targeted therapeutic strategies aimed at restoring ANKH-mediated homeostasis and preventing pathological mineralization.

## Mutation-specific mechanisms in ANKH-related skeletal disorders

### Structural integrity and functional domains of ANKH mutations

**Figure 3:**
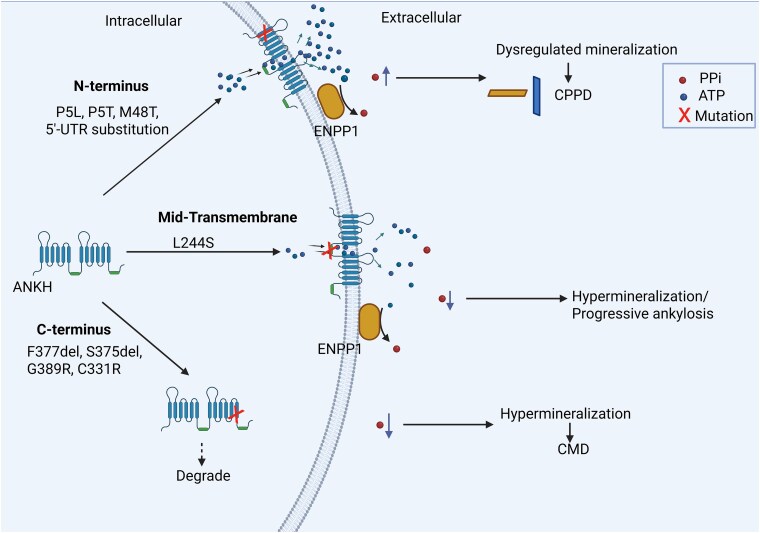
Mutational landscape of progressive ankylosis homolog (*ANKH*) and associated disease phenotypes. Disease-causing *ANKH* mutations cluster in three regions and produce distinct outcomes. N-terminal mutations (P5L, P5T, M48T, and 5′-UTR substitutions) cause local accumulation of extracellular ATP and inorganic pyrophosphate (PPi), driving formation of calcium pyrophosphate crystals and calcium pyrophosphate deposition disease (CPPD). Mid-transmembrane mutations (L244S) reduce ANKH transport activity, lowering extracellular PPi and shifting the balance toward hypermineralization and progressive ankylosis. C-terminal mutations (C331R, S375del, F377del, and G389R) destabilize ANKH and promote its degradation, similarly reducing extracellular PPi and producing the hypermineralization observed in craniometaphyseal dysplasia (CMD). In all cases, ENPP1 acts downstream of ANKH to convert exported ATP into PPi.

The ANKH protein sequence is highly conserved across vertebrate species, with amino acid identity reaching 95.3% between human and chicken and 82% between human and zebrafish, indicating that the protein is under strong evolutionary pressure and tolerates very little sequence variation.^32^ This exceptional conservation reflects the indispensable role of ANKH in normal development, as even single amino acid substitutions are sufficient to cause severe skeletal and metabolic disorders in humans. Indeed, a comprehensive review of existing literature reveals that many of the disease-causing point mutations reported in the human *ANKH* gene, cluster within 3 distinct structural regions of the protein, each associated with specific functional consequences and clinical phenotypes.^31^^,^^62^^,^^63^ Accordingly, these mutations can be broadly categorized by their localization to the N-terminal, mid-transmembrane, and C-terminal domains. Mutations within each of these domains affect protein function differently and lead to distinct pathological outcomes.^8–12^^,^^23^^,^^26–29^^,^^31^^,^^64^^,^^65^ Variants in the N-terminal region, especially within exons 1 and 2, are predominantly associated with CPPD. This autosomal dominant disorder is characterized by CPPD crystal accumulation in cartilage and progressive joint degeneration. These N-terminal mutations usually cause gain-of-function effects, elevating extracellular ATP levels that increase the extracellular PPi levels and subsequently promote crystal formation.^9^^,^^10^^,^^23^^,^^28^^,^^30^ Conversely, mutations located in the C-terminal domain and cytoplasmic loops, primarily within exons 7 and 10, tend to produce loss-of-function outcomes by disrupting proper protein localization or stability, leading to skeletal deformities characteristic of CMD.^8^^,^^10^^,^^11^^,^^26^^,^^27^^,^^29^^,^^31^ A unique case is the L244S mutation, situated in the mid-transmembrane region, which exhibits recessive inheritance and selectively impairs transporter function without affecting protein localization^29^^,^^65^ (Figure 3).

### N-terminal gain-of-function mutations

The N-terminal domain of ANKH, particularly the region encompassing exons 1 and 2, represents a hotspot for gain-of-function mutations that elevate extracellular PPi levels (via increase in ATP transport) and promote CPPD crystal formation. Functional characterization of the P5L (Proline→Leucine) mutation demonstrated approximately 2-fold increase in extracellular PPi compared with the WT protein, resulting from enhanced ENPP1 enzymatic activity that converts extracellular ATP to PPi.^28^ The P5L variant exhibits delayed trafficking to the plasma membrane but remains functionally active once localized. Studies have shown that P5L expression leads to increased extracellular PPi and elevated ENPP1 activity.^23^^,^^28^ Importantly, the functional relationship between ANKH and ENPP1 appears to be substrate-mediated rather than based on direct physical protein–protein interaction. Specifically, ANKH exports ATP into the extracellular environment, where ENPP1, acting as an ectoenzyme at the plasma membrane, hydrolyzes ATP into PPi and AMP. This was elegantly demonstrated in ENPP1-deficient cells, where ANKH overexpression resulted in robust ATP release, but failed to generate extracellular PPi, confirming that ENPP1 activity is an absolute prerequisite for ANKH-dependent PPi formation. To date, no direct physical interaction between ANKH and ENPP1 has been demonstrated through co-immunoprecipitation or yeast 2-hybrid approaches; rather, interactome studies have identified sphingosine kinase 1 and Myb-binding protein 1a as direct ANKH-binding partners.^66^ Therefore, the increased ENPP1 activity observed with N-terminal ANKH mutations such as P5L and P5T likely results from elevated substrate (ATP) availability in the extracellular space rather than from enhanced direct protein–protein interaction. Similar mechanisms were observed for the P5T (Proline→Threonine) variant, which also causes excessive PPi accumulation through increased ANKH protein function. Additional recurrent N-terminal mutations, including M48T (Methionine→Threonine) and the 5′-UTR substitution (–11C>T), further elevate *ANKH* expression and activity, causing greater ATP efflux into the extracellular matrix which increases extracellular PPi.^9^^,^^10^ The resultant extracellular PPi excess inhibits physiological HA crystal formation and favors pathological CPPD crystal deposition within cartilage. Clinically, individuals harboring these mutations typically present in early to mid-adulthood with recurrent attacks of pseudogout, progressive polyarticular arthritis, and extensive chondrocalcinosis predominantly involving the knees, wrists, feet, shoulders, and pubic symphysis. Radiologically, affected joints demonstrate calcifications in both hyaline and fibrocartilage, with symptoms occasionally manifesting as early as the second or third decade of life.^8–10^ Collectively, N-terminal mutations of ANKH therefore represent gain-of-function alterations that disrupt Pi/PPi equilibrium by overstimulating PPi production, underscoring the key role of the N-terminus of ANKH in maintaining healthy mineralization balance.

### Loss-of-function C-terminal mutations

The C-terminal region of ANKH that encompasses exons 7-10, plays a critical role in maintaining proper membrane localization and transport of ATP. Mutations clustered within this region have been primarily associated with CMD, an autosomal dominant skeletal disorder characterized by abnormal craniofacial bone thickening and metaphyseal flaring of long bones.^8–12^ The pathogenic deletion of F377 (Phenylalanine→Deleted) in mice first established a mechanistic association between C-terminal alterations and CMD pathogenesis.^11^^,^^26^^,^^27^ Subsequent studies identified additional variants, including S375del (Serine→Deleted), G389R (Glycine→Arginine), and C331R (Cysteine→Arginine), which disrupt intracellular trafficking by causing mutant ANKH proteins to mislocalize from the plasma membrane into cytoplasmic aggregates.^9^^,^^11^^,^^23^^,^^26^^,^^31^ This mislocalization prevents effective ATP export and reduces the level of extracellular PPi, leading to abnormal mineralization patterns. The resulting imbalance impairs bone remodeling and contributes to the characteristic features of CMD, such as progressive craniofacial hyperostosis, sclerosis of cranial bones, and metaphyseal widening.^28^^,^^29^ Clinically, narrowing of cranial foramina can compress cranial nerves, producing facial palsy, hearing loss, or even optic nerve involvement in severe cases.^11^ These findings support a loss-of-function mechanism whereby reduced levels of ANKH function diminish extracellular PPi and disrupt skeletal mineralization.

### Mid-transmembrane mutation L244S

Alterations within the mid-transmembrane region of ANKH define a distinct pathogenic category with recessive inheritance. The L244S mutation (Leucine→Serine), located in exon 6, causes homozygous individuals to develop a progressive ankylosis-like syndrome characterized by early-onset periarticular soft-tissue calcifications, joint ankylosis, hearing loss, and mild intellectual impairment. Heterozygous carriers generally remain asymptomatic in early life, but may develop mild osteoarthritis later in adulthood, suggesting a dosage-dependent phenotype. Unlike C-terminal mutations that cause protein mislocalization, the L244S variant localizes normally to the plasma membrane yet exhibits severely reduced ATP transporter activity, resulting in lower extracellular PPi, demonstrating that functional ATP transporter defects alone can drive pathological outcomes.^26^^,^^65^

Although considerable progress has been made in identifying disease-causing mutations across the three major structural domains of ANKH, the precise molecular mechanisms by which these mutations drive distinct pathological outcomes remain incompletely understood. Studies have identified mutations in distinct ANKH protein domains that affect protein stability and trafficking, but a comprehensive understanding of how these molecular perturbations contribute to the wide spectrum of clinical mineralization disorders remains lacking. The mechanism by which N-terminal gain-of-function ANKH mutations regulate ENPP1 activity requires further study. Mid-transmembrane domain mutations such as L244S cause functional deficits despite normal membrane localization, indicating unresolved underlying dysfunction. While these C-terminal mutations are considered loss-of-function at the molecular level, disrupting the localization and abolishing canonical ATP export activity of ANKH leads to CMD. The resulting CMD phenotype is unlikely to reflect the loss of ANKH function alone. Notably, *Ank^KO/KO^* and *Ank^ank/ank^* mice, which lack ANKH protein entirely, do not develop hallmark CMD features (mandibular hyperostosis, nasal sinus obliteration, and club-shaped femurs), whereas *Ank^KI/KI^* mice, in which only the C-terminal-mutant protein is expressed, faithfully reproduce these features. This discrepancy may reflect compensatory mechanisms operating in the complete absence of ANKH that are not engaged when a mutant protein is still produced, and/or additional effects of the mislocalized C-terminal mutant on osteoblast and osteoclast biology that are not triggered by the simple absence of ANKH. In support of the latter, *Ank^KI/KI^* mice show disrupted osteoblast and osteoclast function including high bone turnover, paradoxical matrix hypomineralization, and reduced mineralization-gene expression that is not predicted by reduced extracellular PPi alone.^26^^,^^27^ These cellular and molecular abnormalities suggest that the mislocalized mutant protein may interfere with osteoblast and osteoclast differentiation or function in ways that go beyond the consequences of reduced extracellular PPi alone. Further mechanistic studies are needed to clarify how domain-specific ANKH alterations drive distinct pathological outcomes and to guide mutation-specific therapies.

## Model organisms for studying ANKH function and pathology

Domain-specific mutations in ANKH elicit distinct functional and phenotypic consequences, thereby requiring specialized models to reproduce each pathological effect. Mouse models have been invaluable for elucidating the functional outcomes of these mutations, offering critical insights into how they affect mineralization and skeletal phenotypes. While CMD-associated mutations have been modeled in mice and provide valuable insight into skeletal abnormalities, CPPD-associated gain-of-function mutations have not yet been replicated with equivalent fidelity, leaving important aspects of the disease mechanism nebulous.^13^^,^^23–27^^,^^29^^,^^31^^,^^61^^,^^63^ Current murine models used to study ANKH-associated pathologies include the progressive ankylosis (*Ank^ank/ank^*) mouse, which arose from a spontaneous autosomal recessive mutation that introduces a premature stop codon in exon 11 of *Ank* producing a truncated nonfunctional protein,^8^ a separate *Ank* knockout (*Ank^KO/KO^*), which lacks Ank protein expression entirely,^62^ and the *Ank^KI/KI^* knock-in mouse harboring a homozygous Phe377 deletion within the C-terminal region of Ank.^26^^,^^27^

The *Ank^ank/ank^* mouse, a loss-of-function model, recapitulates some CMD-like skeletal abnormalities such as skull thickening, foramen magnum narrowing, and middle-ear bone fusion. However, it fails to model dominant human CMD features like hyperostotic mandibles, nasal sinus obstruction, and flared long bone metaphysis. It also lacks the complex osteoblast and osteoclast dysfunction seen in patients.^8^ The *Ank^KO/KO^* mouse, which lacks Ank protein, displays a phenotype largely indistinguishable from *Ank^ank/ank^* including widespread ectopic joint mineralization, progressive ankylosis, and only subtle CMD-like skeletal features detectable by micro-CT and similarly failing to recapitulate the dominant features of human CMD.^62^ In contrast, the *Ank^KI/KI^* mouse more closely mirrors human CMD phenotypes, displaying hallmark features, such as craniofacial hyperostosis, jawbone enlargement, nasal sinus obliteration, and club-shaped femurs. These characteristics are detectable as early as 1 wk and progressively worsen with age. This phenotypic divergence between *Ank^KI/KI^* and the loss-of-function *Ank^KO/KO^* and *Ank^ank/ank^* models indicates that the mutant protein exerts additional pathological effects beyond simple loss of ANKH function.

Biochemically, serum markers of both bone formation (elevated ALP and procollagen propeptides) and bone resorption (elevated tartrate-resistant acid phosphatase 5b) were raised in *Ank^KI/KI^* mice, indicating a high bone turnover state that drives the progressive craniofacial hyperostosis characteristic of CMD. Paradoxically, despite this excess bone accumulation, the bone matrix was hypomineralized and structurally immature, indicating poor bone quality with likely compromised mechanical strength.^27^ Osteoblasts isolated from *Ank^KI/KI^* mice and cultured to late-stage differentiation showed reduced expression of key mineralization-related genes, including *Mmp13*, *Ocn*, *Runx2*, *Osx*, and *Phex*, alongside a significantly diminished capacity for mineral deposition in culture. This impairment at the cellular level provides a mechanistic basis for the defective bone matrix formation observed in the *Ank^KI/KI^* model and, by extension, in CMD.^26^ The *Ank^KI/KI^* model, does not fully recapitulate all human CMD features, some characteristic long bone metaphyseal widening is less pronounced than in human cases. Also, ectopic calcification and joint stiffness are observed in homozygous *Ank^KI/KI^* mice, but not in heterozygous *Ank^+/KI^* mice, and these features are not typically seen in human CMD, which is inherited as an autosomal dominant condition. Plasma pyrophosphate levels are decreased in these mice, although some compensatory mechanisms are present. Furthermore, the knock-in model represents a homozygous mutation state, whereas most human CMD cases are heterozygous, potentially contributing to phenotypic differences. Overall, while the *Ank^KI/KI^* mouse model provides critical insight into CMD pathology, it falls short of fully mimicking the complex pathophysiology of the human disease, underscoring the need for additional models and approaches.

Other human *ANKH-*associated pathologies such as those linked to CPPD and chondrocalcinosis primarily involve N-terminus mutations that result in gain of function of the protein with impairing ATP transport.^24^ However, no mouse model targeting these N-terminal mutations has yet been developed, despite the high prevalence of CPPD in elderly populations and its considerable morbidity, making this an important gap that warrants the creation of in vivo models to elucidate the distinct pathological mechanisms of these mutations.

Challenges remain in translating results from model organisms to the human disease context. Zebrafish offer distinct advantages, including ease of genetic manipulation, rapid development, and suitability for high-throughput and imaging studies; however, as an evolutionarily distant species, they have physiological differences that limit modeling complex human skeletal diseases.^32^ In contrast, mouse models are evolutionarily closer to humans, making them better suited to study intricate bone remodeling and mineral metabolism, although limitations such as shorter lifespans and the common use of homozygous mutations restrict modeling of long-term disease progression and the heterozygous genetic context seen in most patients.^62^ To overcome these limitations, there is a critical need to develop more advanced mouse models, including conditional or tissue-specific *Ank* mutations, which can more precisely dissect gene-environment interactions and modifier gene effects relevant to ANKH-associated mineralization disorders. By leveraging the complementary strengths of zebrafish and mammalian models, researchers can gain a more comprehensive understanding of the disease spectrum and improve translational relevance.

## Conclusion

A comprehensive understanding of the *ANKH* gene is critical, given its central role in bone mineralization through regulation of extracellular pyrophosphate and mineral deposition within the skeletal matrix. While substantial progress has been made in characterizing ANKH, many important gaps remain in elucidating the full spectrum of its functions, signaling pathways, and the effects of domain-specific mutations. These unresolved questions pose a significant barrier to developing targeted therapies for mineralization disorders linked to ANKH dysregulation. Furthermore, ANKH does not act in isolation; its expression and activity are modulated by a complex network of signaling molecules, miRNAs, and epigenetic regulators, all of which require thorough investigation. Advancing knowledge of these interconnected regulatory mechanisms is essential to deepen insights into bone mineralization and to unlock novel therapeutic strategies for ANKH-associated diseases.

## Data Availability

No new data were generated or analyzed in support of this review. All figures, including the graphical abstract, were created with BioRender.com.
